# Preclinical Demonstration of Synergistic Active Nutrients/Drug (AND) Combination as a Potential Treatment for Malignant Pleural Mesothelioma

**DOI:** 10.1371/journal.pone.0058051

**Published:** 2013-03-06

**Authors:** Viviana Volta, Elia Ranzato, Simona Martinotti, Simone Gallo, Maria Veronica Russo, Luciano Mutti, Stefano Biffo, Bruno Burlando

**Affiliations:** 1 Molecular Histology and Cell Growth Laboratory, San Raffaele Science Institute, Milano, Italy; 2 Dipartimento di Scienze e Innovazione Tecnologica, University of Piemonte Orientale, Alessandria, Italy; 3 Department of General Medicine, Vercelli National Health Trust, Vercelli, Italy; IIT Research Institute, United States of America

## Abstract

Malignant pleural mesothelioma (MPM) is a poor prognosis disease lacking adequate therapy. We have previously shown that ascorbic acid administration is toxic to MPM cells. Here we evaluated a new combined therapy consisting of ascorbate/epigallocatechin-3-gallate/gemcitabine mixture (called AND, for Active Nutrients/Drug). *In vitro* effects of AND therapy on various MPM cell lines revealed a synergistic cytotoxic mechanism. *In vivo* experiments on a xenograft mouse model for MPM, obtained by REN cells injection in immunocompromised mice, showed that AND strongly reduced the size of primary tumor as well as the number and size of metastases, and prevented abdominal hemorrhage. Kaplan Meier curves and the log-rank test indicated a marked increase in the survival of AND-treated animals. Histochemical analysis of dissected tumors showed that AND induced a shift from cell proliferation to apoptosis in cancer cells. Lysates of tumors from AND-treated mice, analyzed with an antibody array, revealed decreased TIMP-1 and -2 expressions and no effects on angiogenesis regulating factors. Multiplex analysis for signaling protein phosphorylation exhibited inactivation of cell proliferation pathways. The complex of data showed that the AND treatment is synergistic *in vitro* on MPM cells, and blocks *in vivo* tumor progression and metastasization in REN-based xenografts. Hence, the AND combination is proposed as a new treatment for MPM.

## Introduction

Malignant pleural mesothelioma (MPM) is a lethal cancer arising from pleura mesothelial cells, showing a close association with previous exposure to asbestos. This tumor is characterized by long latency period (20–30 years) and slow growth which cause late diagnosis, poor prognosis, and limited effective therapies. It has also been suggested that additional factors besides asbestos may play a role in the tumor pathogenesis, such as SV40 infection [1] and genetic predisposition [2].

The problem presented by the disease is exacerbated by the lack of reliable biological markers to be used for early screening, and by its rapid progression following diagnosis, resulting in a median survival time of about 10–12 months [3]. Despite pre-clinical and clinical efforts, there is currently no effective therapeutic approach to MPM. Decisions of carrying out surgery, radiotherapy, chemotherapy or multimodal procedures are taken on a case-by-case basis, and frequently a palliative treatment is the only choice available [4]. Intrusive surgical procedures, based on extrapleural pneumonectomy and pleurectomy, are not suitable for most of the patients due to locally advanced or unresectable disease [5]. Radiotherapy is mainly used as adjuvant therapy following surgery or for symptom relief [6].

In locally advanced or metastatic disease, chemotherapy improves the quality of life and alleviates symptoms. However, the tumor is generally chemoresistant, and most single-agent treatments exhibit low intrinsic activity [7]. Response rates and survival are generally improved by using combination of drugs rather than by single-agent regimens. Combined therapies of cisplatin with antimetabolites are more effective than each single agent alone, and currently represent the standard treatment for MPM [8], [9]. However, patient response rates by far below 50%, and the prognosis remains poor. Other approaches, including gene therapy, vaccines and molecular target therapies are under evaluation, but the need of new therapies for this malignancy is compelling [10].

Among alternative remedies for cancer treatment, there is a growing interest in the preventive action of active nutrients, like vitamins [11]. Several studies suggest that these molecules could also be exploited in a pharmacologic way. Vitamin E analogues, like α-tocopheryl succinate, have been reported to selectively trigger mitochondrial apoptosis in tumor cells [12], while ascorbate, also known as vitamin C, has already been used in clinical trials as an alternative cancer therapy [13], [14]. Based on these data, we decided to investigate the effects of combined active nutrients and pharmaceutical drugs on MPM in a pre-clinical model. Antitumor nutrients are generally better tolerated by the organism than chemotherapeutic drugs, and can both increase the efficacy and allow for lower, safer dosages of these drugs.

In a previous study, we have shown that ascorbate exerts a cytotoxic action on MPM cells, with a lower effect on normal, non-neoplastic mesothelial cells. Ascorbate administration induces extracellular H_2_O_2_ production coupled with an intrinsic higher level of reactive oxygen species (ROS) in MPM cells [15]. These results encouraged us to employ ascorbate in our study, in association with other anti-tumor agents. A series of *in vitro* tests on MPM cells has revealed a synergistic cytotoxicity of ascorbate in combination with the conventional tumor drug gemcitabine, and with the green tea polyphenol epigallocatechin-3-gallate (EGCG) [16].

Gemcitabine is one of the most effective single agents on MPM and is currently used both in combination with chemo/targeted therapy, as a first-line treatment, and as a single agent for second line treatment [17]. EGCG has been found to exert antitumor activity in many cancer models [18], [19]. Even though EGCG is generally known as an antioxidant, mounting evidence points a role in enhancing ROS release, which in turn inhibits tumor growth [20], [21]. In line with these findings, we have previously shown *in vitro* that EGCG is more cytotoxic for MPM cells than for normal mesothelial cells, through a mechanism of action based on extracellular H_2_O_2_ production, Ca^2+^ homeostasis loss, and intracellular ROS increase [22].

In the present preclinical study, we have investigated the *in vitro* interaction of ascorbate with both EGCG and gemcitabine, a triple combined treatment herein defined AND therapy (Active Nutrients/Drug). Thereafter, we have studied the effects of intraperitoneal injections of AND on MPM tumor xenografts growing in the peritoneum of immunodeficient mice.

## Materials and Methods

### Reagents and solutions

(−)-Epigallocatechin-3-gallate (EGCG) was purchased from Cayman Chemical Co. (Ann Arbor, MI, USA); gemcitabine (Gemzar) was from Ely Lilly Italia S.p.A. (Sesto Fiorentino, Italy); L-ascorbic acid (ascorbate), was from Sigma (St. Louis, MO, USA). All other reagents were from Sigma, unless otherwise specified. Ascorbate and EGCG were directly dissolved in culture medium, pH 7.4, while gemcitabine stock solution was prepared in 0.9% NaCl in ultrapure water. For *in vivo* injections, the compounds were dissolved in sterile 0.9% NaCl and the solutions filter-sterilized under hood. L-ascorbic acid was stored as a powder and dissolved immediately prior to use.

### In vitro cell culture

The following human MPM cell lines were available at our laboratory: REN cells are a p53-mutant, inflammatory epithelial subtype [23]; MM98 cells were established from pleural effusion of a sarcomatous MPM [24]; BR95 epithelial cells were obtained from pleural effusions of MPM patients with histologically confirmed malignant mesothelioma [25]; MPP89 are epithelial mesothelioma cells [24]. In addition, epithelial NCI-H28, having a wild-type p53 [26], were purchased from ATCC (cat. no. CRL-5820™, Rockville, MD, USA). Cells were cultured in DMEM supplemented with 10% foetal bovine serum (FBS, Euroclone, Pero, Italy) and 1% antibiotic mixture (Gibco, Invitrogen Life Technologies, S. Giuliano Milanese, Italy), and maintained at 37°C in a humidified atmosphere with 5% CO_2_.

### Cytotoxicity assay

The calcein cytotoxicity assay was carried out by using the lipophilic, nonfluorescent calcein acetoxymethylester (calcein-AM), which penetrates cell membranes and is then cleaved by intracellular esterases, yielding the hydrophilic fluorescent dye. Cells growing in 96-well plates were treated as specified, washed with PBS, and then incubated for 30 min at 37°C with a solution of 2.5 μM calcein-AM in PBS. Plates were read in a fluorescence reader (Infinite 200 Pro, Tecan, Wien, Austria), by using 485-nm excitation and 535-nm emission filters.

### In vitro drug interaction analysis

Dose response curves and IC_50_ values, based on the calcein-AM assay at 48 h, were first derived for single compounds (ascorbate, EGCG, or gemcitabine), as described in Martinotti *et al.* [16]. The concentrations used in these experiments are reported in Table 1. Thereafter, IC_50_ were derived for the AND mixture (ascorbate/EGCG/gemcitabine) by using a constant ratio combination design consisting of serial dilutions of the equipotency concentrations of single compounds (Table 2). After having obtained IC_50_ values, the AND combination was analysed for synergy as described in Martinotti *et al*. [16], by using Chou and Talalay's Combination Index (CI) [27].
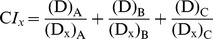
where CI_x_ is the combination index at effect level x% ( =  percent of viability inhibition); (D)_A_, (D)_B_ and (D)_C_ are the doses of drugs A, B and C that combined together inhibit cell viability by x%; (D_x_)_A_, (D_x_)_B_ and (D_x_)_C_ are the doses of drugs A, B and C that inhibit cell viability by x% when used alone. If the CI value is <1,  = 1, or >1, then synergism, additivity or antagonism is indicated, respectively. Variations of drug interaction at different levels of inhibition can be visualized by a plot of CI on the y-axis as a function of effect levels f_a_ on the x-axis (f_a_-CI plot).

**Table 1:** Concentration series used to derive dose-response curves for single compounds.

Compound	Concentrations (µM)								
ascorbate	0	50	100	150	200	250	300	500	1000
EGCG	0	1	5	10	15	20	30	50	
gemcitabine	0	0.1	1	2.5	5	10	20	30	

**Table 2:** Concentration series used to derive dose-response curve for AND in REN cells.

				Dilution	ratios	(µM)		
		EC_50_/48	EC_50_/24	EC_50_/12	EC_50_/6	EC_50_/3	EC_50_	3×EC_50_
Single compounds in mixture	ascorbate	4.75	9.5	19	38	76	228	684
	EGCG	0.42	0.83	1.7	3.33	6.7	20	60
	gem	0.18	0.37	0.74	1.48	2.97	8.9	26.7
Total concentrations in mixture	AND	5.35	10.7	21.4	42.8	85.7	257	771

### Animals and in vivo experiments

Male NOD-SCID CB13 mice (6–8 weeks old) were purchased from Charles River Laboratories Italia Srl (Calco, Italy), and housed for 3–4 days before experiments. Mice were maintained and handled under aseptic conditions, were allowed access to food and water ad libitum, and received i.p. injections of 10×10^6^ REN cells in 1.0 mL of PBS. Mice injected with cells were randomly divided into different treatment groups. Treatments were carried out every 3^rd^ day by i.p. injections. *In vivo* experiments were done in accordance with institutional animal committee guidelines.

### Necropsy and histochemical analyses

At the end of *in vivo* experiments, mice were sacrificed and their abdominal cavity was opened and photographed. Complete necropsy was performed with collection of tumors and major organs and tissues. Necropsied tissues were rapidly frozen in liquid nitrogen and stored at −80°C until use. Part of tumor tissues were fixed in 10% buffered neutral formalin, processed to paraffin and sectioned at 5 µm. Slides were stained with hematoxylin and eosin (H&E) for morphological analysis or used for immuno-histochemistry. Sections were deparaffinized with xylene and graded alcohol, and rehydrated in PBS. Endogenous peroxidases were blocked with 3.0% H_2_O_2_ in PBS. Apoptotic cells were identified on sections using an indirect TUNEL labeling assay (In Situ Cell Death Detection Kit, AP, Roche), according to manufacturer's protocol. Cell proliferation was evaluated by PCNA histochemistry (Abcam, Cambridge, UK), using the Vectastain Elite ABC kit (Vector Laboratories, Burlingame, CA, USA), according to manufacturer's instructions.

### Angiogenesis antibody array

Angiogenesis factors were quantified using the Human Angiogenesis Antibody Array (Panomics, Inc., Redwood City, CA). The array allows for simultaneous detection of 19 factors and provides positive and negative controls. Tumor samples were lysed [28] and hybridized to each membrane of an antibody-sandwich angiogenesis array according to manufacturer's guidelines. Spots were observed and digitized with the Quantity One Imaging system (ChemiDoc XRS, Bio-Rad, Hercules, CA).

### Multiplex analysis of phosphorylated proteins

The phosphorylation of specific signal transduction proteins was analyzed using the Bio-Plex TM bead suspension array system (Bio-Rad), allowing the assay of multiple proteins in a single well. Tumor samples were homogenized in a lysis solution (Bio-Rad), vortexed, centrifuged at 10,000 *g* for 4 min and the supernatant collected. Lysates were adjusted to 1,000 µg/mL protein for use in an assay for 6 different phosphorylated proteins, including Akt (Ser473), Erk 1/2 (Thr202/Tyr204, Thr185/Tyr187), JNK (Thr183/Tyr185), p38 MAPK (Thr180/Tyr182), p70 S6 kinase (Thr421/Ser424), IκBα (Ser32/Ser36). Samples were prepared according to the manufacturer's instructions and sent to Bioclarma srl (Turin, Italy) for fluorescence recording and data analysis.

### Statistics

ANOVA and post hoc tests were carried out using the Instat Software package (GraphPad Software, Inc.). Survival curves were evaluated by the Kaplan-Meier method and compared by the log-rank test [29].

## Results

### AND therapy has a synergistic action in decreasing the viability of MPM cell lines

In a previous study based on the Chou and Talalay's combination index (CI) method [27], we had shown that ascorbate/gemcitabine and ascorbate/EGCG combinations have synergistic cytotoxic activity on REN cells, an established cell model of MPM [16]. We used here the same method to investigate *in vitro* effects of the AND combination (ascorbate/EGCG/gemcitabine) on various MPM cell lines, by using the calcein-AM assay at 48 h. Dose-response curves and IC_50_ values were obtained for each compound alone, and these data were then used to derive dose-response curve and IC_50_ for the triple combination (Table 3). A comparison of the IC_50_s of single compounds obtained on different cell lines showed that ascorbate was the least cytotoxic compound, while gemcitabine was the most cytotoxic one, or was similar to EGCG. An exception was made by NCI-H28, for which a low toxicity of gemcitabine was recorded, possibly depending on the lower growth rate of these cells.

**Table 3:** Values of IC_50_ (µM) determined on different MPM cells by the calcein-AM assay at 48 h.

cell type	ascorbate	EGCG	gemcitabine	AND
REN	228 (202–258)	20 (18–22)	8.9 (6.1–12.8)	38 (26–54)
MM98	47 (36–63)	25 (24–26)	1.1 (0.6–1.9)	13 (12–14)
BR95	189 (175–204)	66 (61–71)	1.6 (0.8–3.1)	83 (77–89)
NCI-H28	706 (645–772)	70 (61–80)	915 (801–1045)	192 (148–249)
MPP89	176 (159–194)	15 (14–16)	23 (7–77)	37 (30–46)

Dose concentration curves showing cell viability (calcein-AM assay) for each single compound and the AND mixture, and for each cell type are shown in Fig. S1 (Supplementary Information).

Dose-response experiments were carried out in duplicate, with a minimum of 6 replicates for each dose. 95% confidence intervals are given in parentheses.

**Figure 1:**
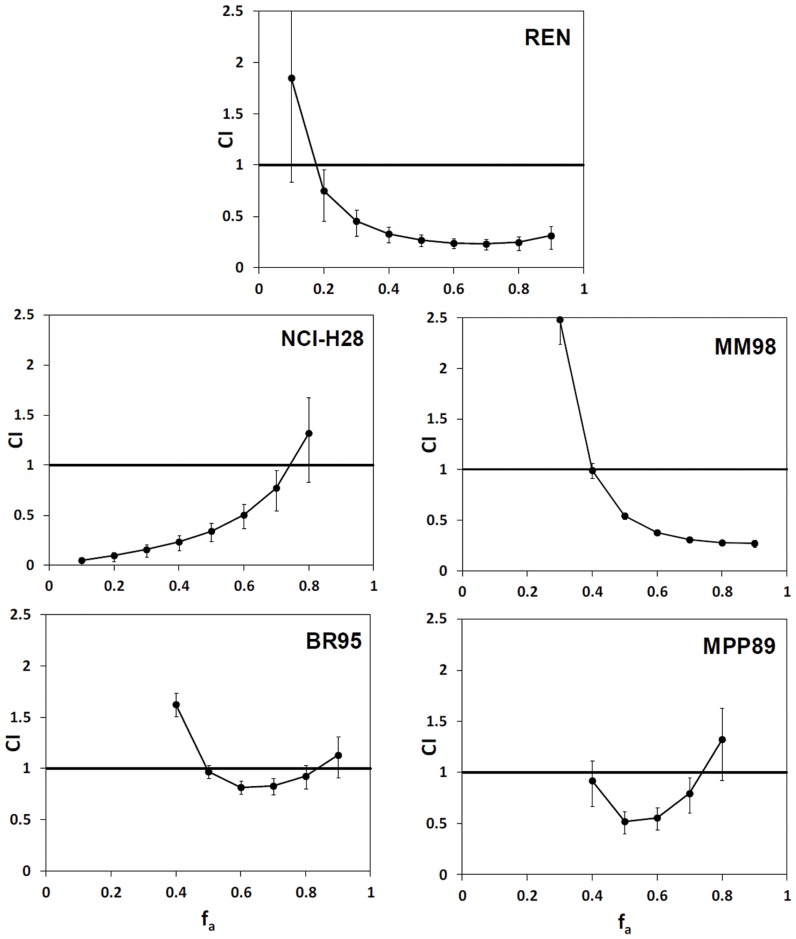
Combination index (CI) of the AND mixture (ascorbate/EGCG/gemcitabine) plotted against the fraction of affected cells (f_a_). The CI values are obtained from quantification of the viability of various MPM cell lines treated with the AND therapy and its single components, as described in the Methods. CI <1,  = 1, and >1, indicate synergy, additivity and antagonism, respectively. For each cell line, 3 independent experiments with 6 replicates each were used.

Data from single and combined treatments were used to assess synergy by Combination Index (CI) analysis, as described in the Methods. The f_a_-CI plots, depicting CI values vs f_a_ (fraction of inhibited viability), showed the occurrence of synergistic effects (CI <1) in all MPM cells, although at variable extents in different cell types (Fig. 1). The strongest synergism at mid-to-high f_a_ was observed in REN cells. We therefore used these cells for *in vivo* experiments, also considering that they have already been documented to be tumorigenic [30].

### AND reduces tumor burden, metastasization and tumor-induced hemorrhage in an MPM mouse model

Pilot tests showed the development of tumor xenografts in all animals injected with REN cells. In final experiments, REN-inoculated animals were randomly stratified into 4 groups of 5 individuals. Each group was exposed to one of the following treatments: (i) gemcitabine alone, used as a reference therapy; (ii) an ascorbate/EGCG mixture, to evaluate the efficacy of active nutrients without a chemotherapeutic drug; (iii) the AND mixture; (iv) a placebo consisting of 0.9% NaCl. Treatments were made every 3^rd^ day, while doses were designed on the basis of literature reports and the results of our preliminary tests. Gemcitabine as single agent was used at 150 mg/kg [31], [32]; ascorbate/EGCG were used at 2,000 mg/kg and 30 mg/kg, respectively [33], [34]; the AND therapy consisted of 2,000 mg/kg ascorbate, 30 mg/kg EGCG, and 100 mg/kg gemcitabine. Such doses were previously tested on tumor – free mice and found to be tolerable for a period of 30 days.

**Figure 2:**
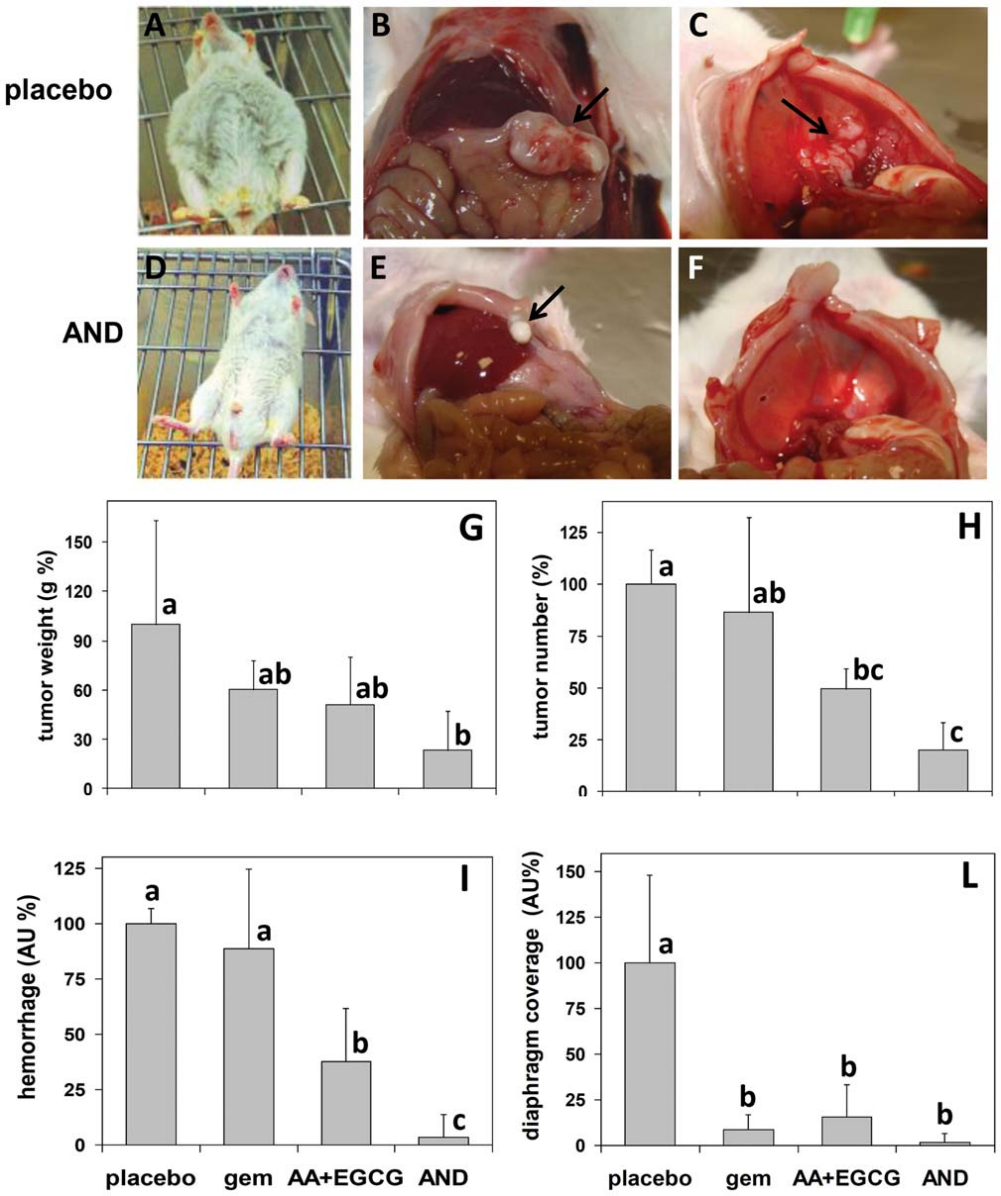
Effects of AND, ascorbate/EGCG (AA+EGCG), and gemcitabine (gem) on MPM tumor development and other symptoms in REN-injected NOD-SCID mice. (A–F) Necropsy examination of mice sacrificed after 30-days treatment. Severe peritoneal hemorrhage (A), big tumor mass (B, arrow) and extended diaphragm coverage (C, arrow) are present in mice treated with placebo. In contrast, AND-treated mice exhibit almost undetectable hemorrhage (D), smaller tumor mass (E, arrow) and lack of diaphragm coverage (F). (G–L) Evaluation of tumor burden and metastasis. Tumor weight (G), tumor number (H), degree of hemorrhage (I), and diaphragm tumor coverage (L) are shown for all the treatments. Data are expressed as means±SD (n = 5) and the means of controls are set to 100%. Letters on bars indicate clustering on the base of statistical differences determined by pairwise comparisons with the Tukey's test. Values labeled with same letters are not statistically different from each other, whereas different letters indicate statistical differences (p<0.01). Values labeled with two letters are not statistically different from either of two other values, but these latter are different from each other.

After 30 days of treatment, animals were sacrificed and necropsied. Untreated mice showed different symptoms of disease including severe ascites, the development of a main tumor, different small tumor nodules at various locations in the peritoneal cavity (mainly on the right kidney, liver and colon), a diffuse metastasization of diaphragm and other alterations such as splenomegaly and intraperitoneal hemorrhage (Fig. 2). The main tumor was located more prevalently on intestinal fat localized on the left side close to the stomach. This was the side subjected to cell injection. All treatments significantly reduced the number and weight of tumor masses, and the degree of hemorrhage and diaphragm tumor coverage, evaluated by arbitrary scores (Fig. 2). However, the strongest reduction in primary tumor development and metastasis was achieved by the AND treatment, along with the total absence of abdominal hemorrhage. Further analyses were therefore focused on this specific treatment.

### AND inhibits cell growth signaling pathways in tumor cells

**Figure 3:**
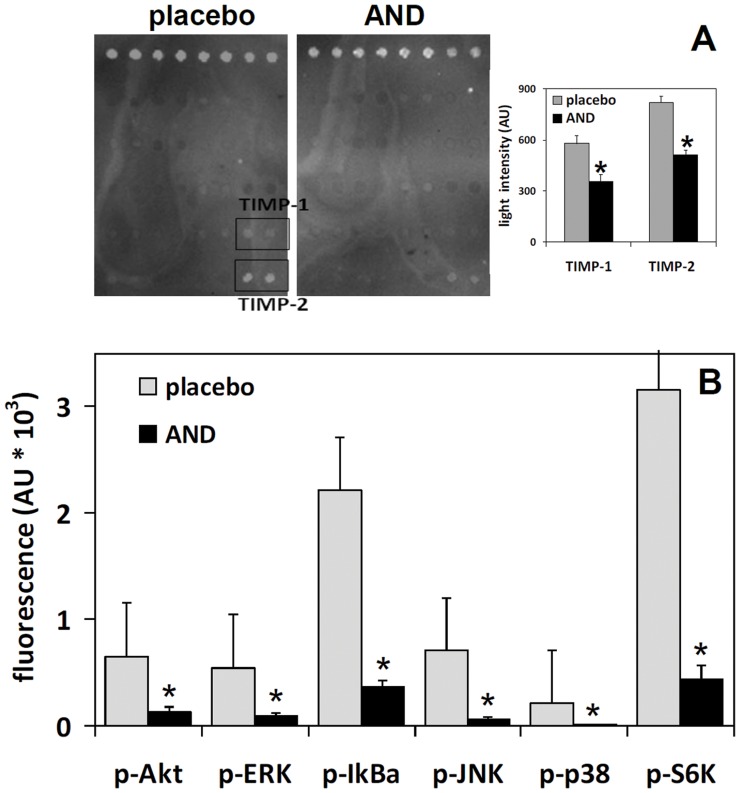
Effects of AND on angiogenesis and on the phosphorylation of signaling proteins in MPM xenografts. (A) Left panels. Detection of angiogenesis factors on membrane antibody arrays by chemiluminescence (one representative experiment is shown). Images are obtained with a CCD camera after 60-s exposures using a Quantity One Imaging system. Each factor is represented by duplicate spots. (A) Right panel. Net light intensity for TIMP-1 and TIMP-2, detected on the basis of gray-scale levels using Quantity One software. Data are means±SD of measurements carried out on 2 membranes with 2 spots each. *  =  p<0.01 according to t test. (B) Phosphorylation status of different cell growth-related proteins evaluated by the Bio-PlexTM multiplex system (see Methods). Fluorescence measurements carried out on 4 different samples are plotted. *  =  p<0.01 according to t test.

The effects of AND on angiogenesis were evaluated by using an array including antibodies for the following factors: angiotensin, VEGF, TNF-α, INF-γ, IL-1α, IL-1β, IL-6, IL-8, IL-12, G-CSF, IP-10, leptin, FGF-α, FGF-β, HGF, PIGF, TGF-β, TIMP-1, and TIMP-2. Tumors excised from mice after 30 days of treatment exhibited low expression levels for most of these factors, and no significant variations induced by AND. The only exception was a reduction of tissue inhibitors of matrix metalloproteinases TIMP-1 and -2 (Fig. 3A). However, a multiplex analysis of signal transduction proteins involved in cell proliferation and growth, including Akt, ERK1/2, IκBα, JNK, p38, and S6K, displayed a general abatement of phosphorylation levels upon AND treatment (Fig. 3B).

### AND increases overall survival of xenograft mice

**Figure 4:**
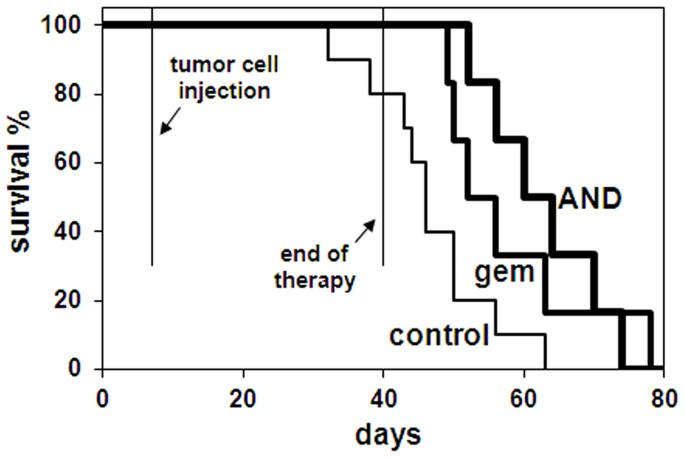
Kaplan-Meier survival curves of immunodeficient mice developing MPM tumor xenografts. Mice were treated with gemcitabine (gem), with the AND mixture, containing a lower dose of gemcitabine (see text), or with placebo (control). By log-rank test analysis, gemcitabine and AND are not significantly different from each other, but either of them induces higher survival rate with respect to placebo (p<0.05).

In a second *in vivo* experiment, to assess animal survival, mice were injected with REN cells as above, and then randomly stratified into 2 groups of 6 animals (treated) and one group of 10 animals (control). Treatments started 7 days after cell injection and were made every 3^rd^ day, as above. Due to an expected longer duration of the survival experiment, a dose-escalating treatment was adopted. Mice were treated with 30 mg/kg gemcitabine as single agent; with an AND mixture of 20 mg/kg gemcitabine, 30 mg/kg EGCG, and 2,000 mg/kg ascorbate; or control treated for 14 days. Thereafter, doses were increased to 50 mg/kg for gemcitabine as single agent, and to 30 mg/kg gemcitabine, 30 mg/kg EGCG, and 2,000 mg/kg ascorbate for AND, for an additional period of 14 days. Mice died spontaneously or were sacrificed to avoid excessive suffering (mainly due to heavy intraperitoneal hemorrhage with abdominal enlargment). Kaplan-Meier curves and the log-rank test indicated a significant increase of overall survival in animals treated with AND or gemcitabine, as compared to control-treated (Fig. 4). Most AND-treated animals survived for a longer time than the gemcitabine-treated ones, but the trend was not statistically significant. However, AND contained lower dosage of gemcitabine.

### AND inhibits tumor cell proliferation

**Figure 5:**
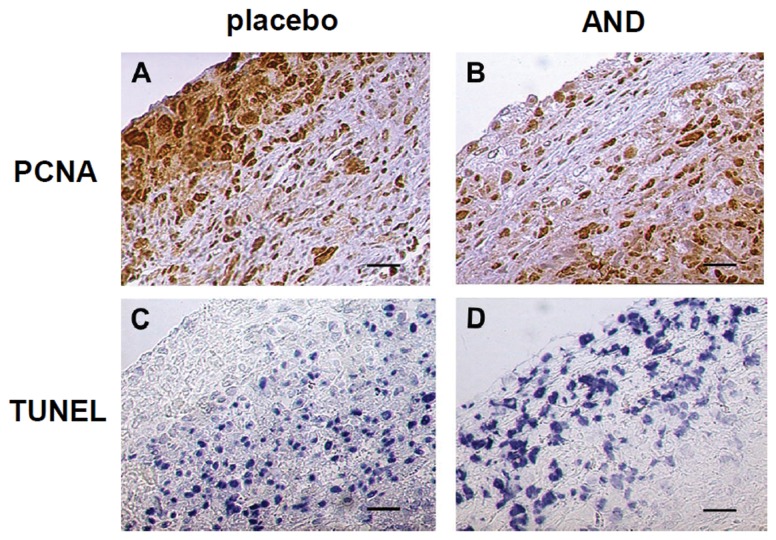
Effects of AND on cell proliferation and apoptosis in tumor xenografts. Tumors were dissected and processed by histochemical techniques as described in the Methods. PCNA staining shows high proliferation rate in the superficial area of control tissue section (A), that is not evident in the AND-treated one (B). In contrast, TUNEL assay shows apoptotic cells in the superficial area of AND-treated tissue (D), but not of control one (C). Bar 50 µm.

Tissues from xenograft tumors were excised, formalin fixed, paraffin embedded, and stained for markers of cell proliferation and apoptosis. The histology of REN-derived tumor masses revealed a more densely cellularized superficial sheath surrounding a central mass of fibrous tissue with scattered cells. According to such tissue organization, histochemistry revealed a prevalence of cell proliferation at the tumor surface (according to PCNA staining, Fig. 5A), whereas the internal portion was characterized by a prevalence of apoptosis (according to TUNEL assay, Fig. 5C). These data suggest that tumor growth occurs mainly at the surface while more internal portions progressively transform into fibrous tissue, possibly due to hypoxic conditions. In AND-treated tumors, staining patterns were drastically different. Surface layers showed a drop in cell proliferation accompanied by an increase in apoptosis (Fig. 5B, D). This suggests that AND treatment diffusing from the surface toward the interior of tumor mass blocks cancer proliferation.

## Discussion

Here we show that a triple combined treatment based on EGCG, ascorbate and gemcitabine (AND therapy) reduces mesothelioma growth and metastasization. Due to the lack of side effects, we propose that this combined therapy should be evaluated in other preclinical and clinical models.

Ascorbate, a known active nutrient, is well tolerated by the human body and exerts antitumor effects both *in vitro* and *in vivo* [35], [36]. We therefore used ascorbate on MPM cells *in vitro*, and showed selective cytotoxicity due to a maladaptive redox mechanism of these cells causing strong oxidative stress [15]. However, given the chemoresistance of MPM, our therapeutic goal was to combine ascorbate with other drugs in order to maximally strengthen the final effect through a synergistic mixture.

In an *in vitro* screening of various ascorbate/drug combinations, two compounds showed synergistic effects against MPM cells, viz. the standard antitumor gemcitabine, and EGCG, an active nutrient with antitumor properties [16]. The next step has been to combine ascorbate with these two compounds in the triple AND treatment and achieve a preclinical assessment of its feasibility as an anti-MPM therapy. Ascorbate and EGCG are mainly known as antioxidants from a nutritional point of view, but investigations regarding their antitumor properties have also pointed out pro-oxidant properties [13], [15], [20]. This study provided a confirmation of our previous results, showing that when EGCG and gemcitabine are combined together with ascorbate to form the AND mixture, a synergistic effect is obtained.

For preclinical investigations we used a murine model of MPM, developed by injecting tumorigenic REN cells within NOD-SCID immunodeficient mice, a strain that is widely used in tumor biology and xenograft research [37]. This model showed various symptoms of disease at necropsy, including main tumor masses close to the site of injection, secondary tumor nodules at various abdominal locations and on the diaphragm, and acute abdominal hemorrhage.

In previous studies, different combinations of our active nutrients with conventional antitumor drugs have been reported. It has been shown that ascorbate increases the effects of arsenic trioxide, doxorubicin, cisplatin and paclitaxel on human breast cancer cells [38], [39], of 5-fluorouracil and cisplatin on esophageal cancer cells [40], and of gemcitabine in a preclinical model of pancreatic cancer [41]. EGCG reportedly sensitized breast cancer cells to paclitaxel [42], overcame resistance to etoposide-induced apoptosis [43], and increased apoptosis rates induced by gemcitabine, mitomycin C, or 5-fluorouracil in cholangiocarcinoma cells [44].

In our study, the synergistic power of the AND mixture was firstly demonstrated by cytotoxicity and combination index analysis. Data from REN cells indicate that at a 50% effect level, induced by AND at 42.8 µM, the mixture is synergistic with individual concentrations of ascorbate, EGCG, and gemcitabine at 38, 3.3, and 1.48 µM, respectively. All these figures are about sevenfold lower than those inducing the same effect when the compounds are used alone. This results in concentrations compatible with those achieved in human plasma [45]–[47], while it is likely that these EGCG levels could be tolerated by humans, as suggested by safety assessment on rodents [48].

For *in vivo* experiments, we have chosen an intraperitoneal xenograft mouse model of MPM. This model has already been used in various studies, [30], [49], [50] showing that it is well-suited for mesothelioma research, and may be useful for evaluating novel antitumor treatments *in vivo*. Also, as reported in the Results, we have selected doses for each single component of the injected mixtures previously determined to be safe to animals. This was also confirmed by our preliminary tests.

By using this experimental design, strong anti-MPM effects of AND have emerged at different observational levels. At population level, Kaplan-Meier analysis showed significant survival increase for gemcitabine-treated mice, but an identical result was achieved with AND treatment, where gemcitabine dosage was about one third lower. At organism level, the AND mixture inhibited tumor onset, metastasis and tumor-related symptoms like internal hemorrhage. In this respect, gemcitabine alone failed to reduce internal hemorrhage, possibly due to adverse collateral effects, thus further arguing for the superiority of the AND therapy. The lower dose of gemcitabine in the AND therapy seems to reduce the drug's toxic effects, and indicates that the combined therapy fits the goal of this study. At the cellular level, there was a shift from cell proliferation to apoptosis in the outermost layer of tumor mass, concomitantly with the inactivation of kinases involved in cell growth. Conversely, angiogenesis factors did not seem to be particularly expressed in MPM tumor xenografts, or specifically targeted by the treatment.

Taken together, our data indicate that the AND treatment inhibits tumor growth and invasiveness. The mechanism of action is likely to involve redox processes, as suggested by our previous data about the effects of ascorbate or EGCG on MPM cells [15], [22]. However, the AND synergism also indicates that the combined effect is not a mere sum of its single constituents.

In conclusion, in this study we have proposed a new possible therapy for MPM, based on a novel, synergistic combination of active nutrients/drug, all used at pharmacological doses. Data provided the following pieces of evidence.

1. The AND treatment showed *in vitro* synergistic anti-MPM activity.
2. *In vivo* experiments on a murine MPM model showed that AND vigorously inhibited the development of disease and exerted a better therapeutic action at reduced dosages of gemcitabine.
3. Data indicated a shift from cell proliferation to apoptosis, blocking tumor growth and invasiveness.

Based on these data, we propose the AND therapy as a possible new treatment to be tested on MPM patients in clinical trials.

## Supporting Information

Figure S1 **Dose concentration curves showing cell viability (calcein-AM assay) for each single compounds and the AND mixture, and for each cell type. Vertical dotted line: IC50; vertical continuous line: IC05. Horizontal lines: 95% CI.**
(DOCX)
