# Supplementary figures and images for: Preclinical Demonstration of Synergistic Active Nutrients/Drug (AND) Combination as a Potential Treatment for Malignant Pleural Mesothelioma
Source: PLoS One. 2013 Mar 6;8(3):e58051. doi: 10.1371/journal.pone.0058051 (PMC3590277; doi:10.1371/journal.pone.0058051)

| **Cell line** | **AA** | | **EGCG** | | **Gemcitabine** | | **AND** | |
| --- | --- | --- | --- | --- | --- | --- | --- | --- |
| **REN** | 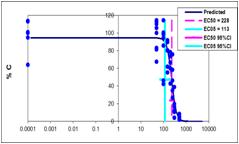 | | 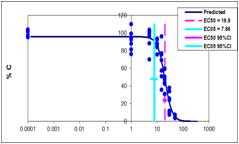 | | 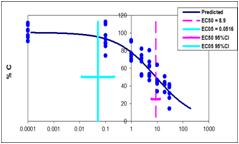 | | 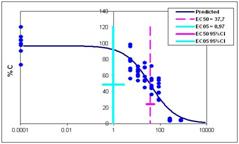 | |
| **MM98** | 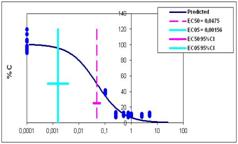 | | 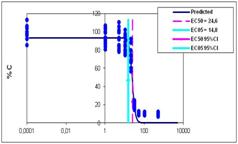 | | 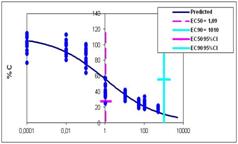 | | 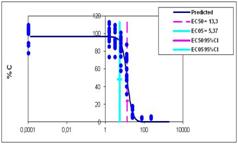 | |
| **BR95** | 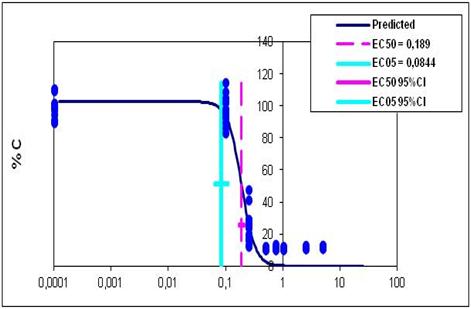 | 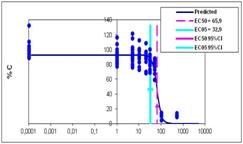 | | 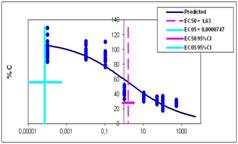 | | 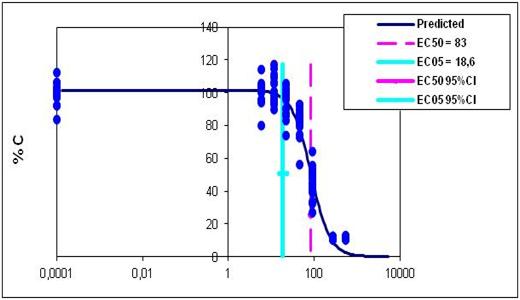 | |
| **NCI-H28** | 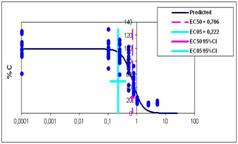 | | 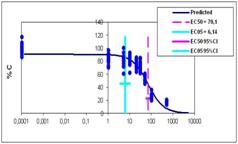 | | 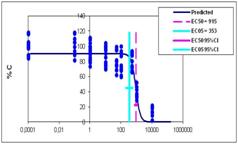 | | 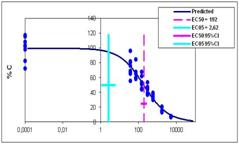 | |
| **MPP89** | 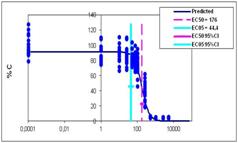 | | 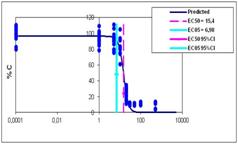 | | 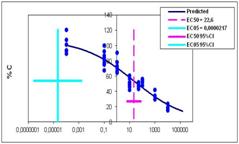 | | 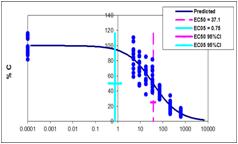 | |

Supplement: Figure S1 — Dose concentration curves showing cell viability (calcein-AM assay) for each single compounds and the AND mixture, and for each cell type. Vertical dotted line: IC50; vertical continuous line: IC05. Horizontal lines: 95% CI. (DOCX) [file pone.0058051.s001.docx]
